# Patientenaufkommen, Diagnosen und Verletzungsmechanismen eines überregionalen Traumazentrums mit Beginn der COVID-19-Pandemie im Vergleich zum Mittelwert der 3 Vorjahre: Eine retrospektive, epidemiologische Auswertung von 4967 Patienten

**DOI:** 10.1007/s00113-020-00894-4

**Published:** 2020-09-28

**Authors:** T. Graulich, J. Gerhardy, P. Gräff, M. Örgel, T. Omar Pacha, C. Krettek, C. Macke, E. Liodakis

**Affiliations:** Klinik für Unfallchirurgie, Medizinische Hochschule Hannover, Carl-Neuberg-Str. 1, 30625 Hannover, Deutschland

**Keywords:** COVID-19/SARS-CoV-2, Notaufnahme, Gereatrische Fraktur, Patientenaufkommen, Verletzungsmechanismen, COVID-19/SARS-CoV-2, Emergency admission, Gereatric fracture, Patient volume, Injury mechanisms

## Abstract

**Hintergrund:**

Im Rahmen der COVID-19-Pandemie wurden zur Reduktion der Infektionszahlen politische Entscheidungen getroffen, die die soziale Interaktion reduzieren sollen. Ziel hierbei war das Schaffen von Kapazitäten zur innerhospitalen Versorgung der erkrankten Patienten.

**Ziel der Arbeit:**

Ob seit Beginn der getroffenen Maßnahmen auch eine Reduktion des unfallchirurgischen Patientenaufkommens im Vergleich zum Mittelwert der 3 Vorjahre zu beobachten ist, sollte geprüft werden.

**Material und Methoden:**

Es wurden retrospektiv alle Patienten, die sich in unserer unfallchirurgischen Notaufnahme im Zeitraum vom 01.03.2020 bis 15.04.2020 vorgestellt haben, mit dem Mittelwert der Patienten der 3 Vorjahre 2017–2019 verglichen. Es wurden Alter der Patienten, Vorstellungszeitpunkt, Diagnosen, Verbleib der Patienten, stationär oder ambulant, Anzahl und Dauer der operativen Versorgung und benötigte Kapazität auf Normal- und Intensivstation erhoben. Der Verletzungsmechanismus wurde ebenfalls untersucht.

**Ergebnisse:**

Es wurden insgesamt 4967 Patienten im Beobachtungszeitraum vom 01.03.–15.04. eingeschlossen. Im Schnitt der 3 Vorjahre wurden insgesamt 1348 Patienten, d. h. pro Tag 29,3 Patienten vorstellig. Im Jahr 2020 wurden insgesamt 923, d. h. pro Tag 20 Patienten vorstellig (*p* < 0,01).

Im Schnitt der Vorjahre wurden 227 (24,6 %) im Vergleich zu 2020 311,5 (23,1 %) stationär aufgenommen. Im Schnitt der Vorjahre resultierten 143 im Vergleich zu 2020 mit 136 Operationen hieraus. Die stationären Tage waren von 2442 im Schnitt der Vorjahre, im Jahr 2020, mit 1172 Tagen um 52,1 % reduziert. Die Anzahl der Tage auf der ICU betrug im Mittel der Vorjahre 450 Tage und im Jahr 2020 303 Tage (−32,7 %).

**Diskussion:**

Das Patientenaufkommen in der unfallchirurgischen Notaufnahme war im Beobachtungszeitraum im Jahr 2020 im Vergleich zum Mittelwert der 3 Vorjahre deutlich reduziert. Hierdurch sind direkt Ressourcen für die Betreuung von COVID-19-Patienten frei geworden.

## Abstract (en)

**Background:**

As part of the COVID-19 pandemic, political decisions were made to reduce social interaction and to reduce the number of infections. The aim was to create capacities for the in-hospital care of the patients.

**Objective:**

The aim of the study was to check whether a reduction in the number of trauma patients compared to the mean of the previous 3 years could be observed.

**Material and methods:**

We retrospectively analyzed all patients who presented in the emergency admission from 1 March to 15 April 2020 with the mean of the patients from the previous 3 years 2017–2019. The age of the patients, time of presentation, diagnoses, whereabouts of the patients, inpatient or outpatient, number and duration of the operative care and required capacity on the normal ward and intensive care units (ICU) were recorded. The injury mechanism was also examined.

**Results:**

A total of 4967 patients between 1 March and 15 April were included. On average over the 3 previous years, a total of 1348 patients, i.e. 29.3 patients per day were counted in our emergency room. In 2020 a total of 923, i.e. 20 patients per day (*p* < 0.01) were counted.

On average 227 (24.6%) were admitted to hospital compared to 311.5 (23.1%) in 2020. On average 143 operations were performed compared to 136 in 2020. The days on the ward were reduced from 2442 on average for the previous years, in 2020 to 1172 days by 52.1% (*p* < 0.01). The number of days on the ICU was 450 days on average in previous years and 303 days in 2020 (−32.7%, *p* < 0.01).

**Conclusion:**

The number of patients in the emergency admission was significantly reduced in the observation period in 2020 compared to the mean of the previous 3 years. This directly made resources available for the care of COVID-19 patients.

## Einleitung

Erstmals wurde in China in der Region Wuhan im Dezember 2019 eine neue Atemwegserkrankung beobachtet [1]. Die Ausbreitung der Atemwegserkrankung erfolgte durch Infektion mit dem „severe acute respiratory syndrome coronavirus 2“ (SARS-CoV-2) [2, 3]. Die Erkrankung durch Infektion mit dem SARS-CoV‑2 wird als „coronavirus disease 2019“ (COVID-19) bezeichnet [4]. Um die Infektionszahlen zu reduzieren, wurden in Deutschland Maßnahmen getroffen, die die gesellschaftlichen Interaktionen reduzieren. Erfahrungen mit dem H1N1-Virus zeigten, dass Massenveranstaltungen als eine der Hauptursachen der Verbreitung von Virusinfektionen angesehen werden können [5]. Das Aufkommen des SARS-CoV‑2 in China in 2019/2020 und die anschließende Ausbreitung wurden schließlich durch die WHO zur COVID-19-Pandemie erklärt. Die daraus resultierenden politischen Entscheidungen mit Maßnahmen, wie der Absage von Massenveranstaltungen, gezielter Schließung der Schulen und des Einzelhandels, Produktionsstopps größerer Betriebe und einer Ausgangsbeschränkung mit dem Erlass, Ansammlungen von mehr als 2 Personen zu meiden, dienten der Reduktion der Verbreitung von COVID-19. Ziel der Reduktion der Infektionszahlen und der Verlangsamung der Infektionsraten war, die Mortalität der Erkrankung zu senken und die Belastung des Gesundheitssystems zu reduzieren, um weiterhin eine Individualversorgung gewährleisten zu können. In der Region Hannover wurde am 01.03.2020 der erste Patient mit SARS-CoV‑2 beobachtet. Die Erkrankung rückte so nicht nur in das lokal-gesellschaftliche Bewusstsein, sondern auch weiter in den politischen Vordergrund. In der Medizinischen Hochschule Hannover (MHH) wurde am 14.03.2020, als Reaktion auf die Empfehlung der Landesregierung vom Vortag, eine Krankenhauseinsatzleitung (KEL) implementiert, um dynamisch auf das sich täglich ändernde Infektionsgeschehen reagieren und die Abläufe zentral koordinieren zu können. Hieraus erging u. a. mit Wirkung zum 14.03.2020 die Einstellung des elektiven OP-Programms.

Die Unfallchirurgie/Orthopädie, die in einem großen Anteil an der Notfallversorgung der in der Notaufnahme vorstellig werdenden Patienten beteiligt ist, benötigt einen nicht unerheblichen Anteil der insgesamt vorhandenen Ressourcen. Für den Großraum München konnte gezeigt werden, dass das Patientenaufkommen der Unfallchirurgie/Orthopädie ca. 43 % des gesamten Patientenaufkommens der Notaufnahmen ausmacht [6].

Ob die getroffenen politischen Maßnahmen auch einen Einfluss auf das Patientenaufkommen der in der Notaufnahme vorstellig werdenden Patienten hat, und ob sich die Art der Verletzungen sowie die Unfallmechanismen unterscheiden, bleibt unklar.

Daher sollten folgende Fragen geklärt werden:

1. Kam es im März/April 2020 zu einem Rückgang des Patientenaufkommens in der unfallchirurgischen Notaufnahme im Vergleich zum Mittelwert der 3 vorhergehenden Jahre?
2. Kam es zu veränderten Verletzungsaufkommen und Unfallmechanismen?
3. War dies mit einer direkten Reduktion von stationärer Aufenthaltsdauer, Operationsdauer und Intensivaufenthalt verbunden?

## Material und Methoden

### Untersuchungsgebiet und Klinikdaten

Die Stadt Hannover mit 556.695 Einwohnern (Stand 31.12.2019) auf einer Fläche von 20.415 ha (Stand 01.01.2020) umfasst 12 Kliniken mit insgesamt 4795 Krankenhausbetten, wovon 1520 Betten auf die MHH entfallen (Statistischer Bericht Niedersachsen; Landesamt für Statistik, Stand 2017) [7]. Die Unfallchirurgie der MHH gilt als überregionales Traumazentrum und versorgt Verletzungen jeden Schweregrads.

### Datenerfassung und -auswertung

Es handelt sich um eine vergleichende, epidemiologische Studie. Die Daten wurden retrospektiv im Zeitraum vom 01.03.2020 bis 15.04.2020 erfasst. Als Referenzwert galt der Mittelwert der 3 Vorjahre 2017–2019 im selbigen Zeitraum, vom 01.03.–15.04. des jeweiligen Jahres.

Das Studienprotokoll wurde der Ethikkommission der MHH vorgelegt, die keine Beanstandung hatte und die Beratungspflicht aussetzte (Nr. 9012_BO_K_2020).

Alle Patienten, die sich in oben genannten Zeitraum mit einem muskuloskeletalen Beschwerdebild in unserer interdisziplinären Notaufnahme vorgestellt haben und unfallchirurgisch versorgt wurden, wurden in die Studie eingeschlossen. Es wurden anonymisiert Datum und Uhrzeit der Vorstellung erfasst. Ebenso wurde die Diagnose erfasst. Die Klassifikation der Diagnosen basierte auf der Internationalen Statistischen Klassifikation der Krankheiten und verwandter Gesundheitsprobleme (10. Revision German Modification (ICD-10-GM) Version 2020) [8]. Entsprechend den Pathologien für das Fachgebiet Unfallchirurgie und Orthopädie wurden die Kapitel XIII (Krankheiten des Muskel-Skelett-Systems und des Bindegewebes) und XIX (Verletzungen, Vergiftungen und bestimmte andere Folgen äußerer Ursachen) untersucht (Tab. 1).

**Table 1:** 

Unfallchirurgische/orthopädische Krankheitsbilder	MW 17–19 [*n*]	Diagnosen 20 [*n*]	MW 17–19 [%]	Diagnosen 20 [%]	Dif. [%]
Verletzungen, Kopf [S00–S09]	286,6	189	20,8	20,3	−0,5
Verletzungen des Knies und des Unterschenkels [S80–S89]	130	93	9,5	9,9	0,4
Verletzungen der Knöchelregion und des Fußes [S90–S99]	165,3	81	12,1	8,7	−3,4
Verletzungen, Ellenbogengelenk/Unterarm [S50–S59]	84	78	6,2	8,4	2,2
Sonstige Diagnosen	75,6	75	5,5	8	2,5
Verletzungen der Hüfte und des Oberschenkels [S70–S79]	61	57	4,4	6,1	1,7
Verletzungen des Thorax [S20–S29]	79	53	5,8	5,7	−0,1
Verletzungen, Schulter/Oberarm [S40–S49]	81,6	49	5,9	5,3	−0,6
Verletzungen, Handgelenk/Hand [S60–S69]	130	48	9,4	5,2	−4,2
Verletzungen nicht näher bezeichneter Teile des Rumpfes, der Extremitäten oder anderer Körperregionen [T00–T14]	17,3	48	1,2	5,2	4
Komplikationen bei chirurgischen Eingriffen und medizinischer Behandlung, anderenorts nicht klassifiziert [T80–T88]	53,6	38	3,9	4,1	0,2
Verletzungen des Halses [S10–S19]	46,6	30	3,4	3,2	−0,2
Verletzungen des Abdomens, des Lumbosakralgelenks, der LWS und des Beckens [S30–S39]	49	28	3,6	3	−0,6
Krankheiten der Weichteilgewebe [M60–M79]	38,3	24	2,8	2,6	−0,2
Arthropathien [M00–M25]	50	20	3,6	2,1	−1,5
Krankheiten der Wirbelsäule und des Rückens [M40–M54]	9,3	10	0,7	1,1	0,4
Osteopathien und Chondropathien [M80–M94]	7	5	0,5	0,5	0
Bestimmte Frühkomplikationen eines Traumas [T79]	2,6	3	0,2	0,3	0,1
Verbrennungen oder Verätzungen [T20–T32]	0,6	1	0	0,1	0,1
Toxische Wirkungen von vorwiegend nicht medizinisch verwendeten Substanzen [T51–T65]	1,6	1	0,1	0,1	0
Sonstige Krankheiten des Muskel-Skelett-Systems und des Bindegewebes [M95–M99]	0	1	0	0,1	0,1
Folgen des Eindringens eines Fremdkörpers durch eine natürliche Körperöffnung [T15–T19]	1,3	0	0,1	0	−0,1
Erfrierung [T33–T35]	0	0	0	0	0
Vergiftungen durch Arzneimittel, Drogen und biologisch aktive Substanzen [T36–T50]	0	0	0	0	0
Sonstige und nicht näher bezeichnete Schäden durch äußere Ursachen [T66–T78]	3	0	0,2	0	−0,2
Sonstige Komplikationen eines Traumas, anderenorts nicht klassifiziert [T89]	1,3	0	0,1	0	−0,1
Folgen von Verletzungen, Vergiftungen und sonstigen Auswirkungen äußerer Ursachen [T90–T98]	0	0	0	0	0
Systemkrankheiten des Bindegewebes [M35–M36]	0	0	0	0	0
*Summe*	*1374,63*	*932*	*100*	*100*	–

*MW* Mittelwert, *n* Anzahl, *%* Prozent, *Dif.* Differenz 2020 zum Mittelwert der 3 Vorjahre

Da die ICD-10-Klassifikation u. a. die Verletzungen nach Körperregionen klassifiziert, jedoch aus dem Code kein direkter Aufschluss über die Verletzungsschwere ergeht, wurden parallel Frakturen entsprechend den einzelnen Körperregionen zusammengefasst (Tab. 2). Ferner wurden offene Wunden entsprechend den Körperregionen zusammengefasst (Tab. 3).

**Table 2:** 

Unfallchirurgische/orthopädische Krankheitsbilder	MW 17–19 [*n*]	Frakturen 20 [*n*]	MW 17–19 [%]	Frakturen 20 [%]	Dif. [%]
Verletzungen, Kopf [S00–S09]	26,6	24	7,5	9,2	1,7
Verletzungen des Halses [S10–S19]	7	10	1,9	3,8	1,9
Verletzungen des Thorax [S20–S29]	33,3	21	9,4	8,1	−1,3
Verletzungen des Abdomens, des Lumbosakralgelenks, der LWS und des Beckens [S30–S39]	21	19	5,9	7,3	1,4
Verletzungen, Schulter/Oberarm [S40–S49]	45,6	24	12,8	9,2	−3,6
Verletzungen, Ellenbogengelenk/Unterarm [S50–S59]	53,6	55	14,9	21,2	6,3
Verletzungen, Handgelenk/Hand [S60–S69]	51	17	14,3	6,5	−7,8
Verletzungen der Hüfte und des Oberschenkels [S70–S79]	35,6	40	9,9	15,4	5,5
Verletzungen des Knies und des Unterschenkels [S80–S89]	40,3	36	11,3	13,9	2,6
Verletzungen der Knöchelregion und des Fußes [S90–S99]	45,3	14	12,1	5,4	−6,7
*Summe*	*359,3*	*260*	*100*	*100*	–

*MW* Mittelwert, *n* Anzahl, *%* Prozent, *Dif.* Differenz 2020 zum Mittelwert der 3 Vorjahre

**Table 3:** 

Unfallchirurgische/orthopädische Krankheitsbilder	MW 17–19 [*n*]	Wunden 20 [*n*]	MW 17–19 [%]	Wunden 20 [%]	Dif. [%]
Verletzungen, Kopf [S00–S09]	65,6	40	52,3	54,8	2,5
Verletzungen des Halses [S10–S19]	0,3	1	0,2	1,4	1,2
Verletzungen des Thorax [S20–S29]	0,3	0	0,2	0	−0,2
Verletzungen des Abdomens, des Lumbosakralgelenks, der LWS und des Beckens [S30–S39]	2	1	1,7	1,4	−0,3
Verletzungen, Schulter/Oberarm [S40–S49]	1,3	0	1	0	−1
Verletzungen, Ellenbogengelenk/Unterarm [S50–S59]	7,6	7	6	9,6	3,6
Verletzungen, Handgelenk/Hand [S60–S69]	26,3	3	20,9	4,1	−16,8
Verletzungen der Hüfte und des Oberschenkels [S70–S79]	2,6	5	2,2	6,8	4,6
Verletzungen des Knies und des Unterschenkels [S80–S89]	12,6	11	10,1	15,1	5
Verletzungen der Knöchelregion und des Fußes [S90–S99]	6,6	5	5,4	6,8	1,4
*Summe*	*125,2*	*73*	*100*	*100*	–

*MW* Mittelwert, *n* Anzahl, *%* Prozent, *Dif.* Differenz 2020 zum Mittelwert der 3 Vorjahre

Der Unfallmechanismus wurde ermittelt und in folgende Gruppen zusammengefasst: Verletzung im häuslichen Umfeld, Verletzung im BGlichen Umfeld, atraumatische Beschwerden, Verletzungen im Pflegeheim, Verletzungen beim Sport, Verletzungen durch Gewalteinwirkung, Verletzungen durch Heimwerkerarbeiten, Verletzungen in der Schule (ebenfalls BGlich), Verletzungen nach VU, Verletzungen, sonstige, Verletzungen auf der Straße (privat) (Tab. 5).

Alle Patienten, die auf eine Normalstation oder Intensivstation aufgenommen wurden, wurden als solche klassifiziert. Alle übrigen Patienten wurden als ambulante Patienten klassifiziert. Bei Patienten, die stationär aufgenommen wurden, wurde die Dauer des stationären Aufenthalts bestimmt. Ferner wurde, sofern zutreffend, die Dauer des Intensivaufenthalts bestimmt, um die benötigte Intensivkapazität beurteilen zu können. Des Weiteren wurden diese Patienten hinsichtlich einer etwaigen Operation beurteilt. Sofern sie operiert wurden, wurde die Dauer der Operation (Schnitt-Naht [Minuten]) dokumentiert, um die benötigte OP-Kapazität zu beurteilen (Tab. 4).

**Table 4:** 

Unfallmechanismus	Ø 17–19 [*n*]	Ø 17–19 [%]	2020 [*n*]	2020 [%]	Dif. [%]
Verletzung im häuslichen Umfeld	267	21,2	259	27,8	6,6
Verletzung im BGlichen Umfeld	227	18,1	160	17,1	−1
Verletzungen, sonstige	214	17,1	156	16,7	−0,4
Verletzungen beim Sport	164	13,1	73	7,8	−5,3
Atraumatische Beschwerden	19	1,5	62	6,7	5,2
Verletzungen nach VU	73	5,8	62	6,6	0,8
Verletzung im Pflegeheim	64	5,1	53	5,7	0,6
Verletzungen durch Gewalteinwirkung	44	3,5	36	3,9	0,4
Verletzungen durch Heimwerkerarbeiten	25	2	35	3,8	1,8
Verletzungen auf der Straße (privat)	113	9	32	3,4	−5,6
Verletzungen in der Schule (ebenfalls BGlich)	45	3,6	5	0,5	−3,1
Summe	1255	100	933	100	–

*Ø* Mittelwert, *n* Anzahl, *Dif.* Differenz 2020 zum Mittelwert der 3 Vorjahre

Die statistische Auswertung erfolgte mit IBM SPSS Statistics® Version 25 (SPSS Inc., Chicago, Il, USA). Zunächst wurden die Daten auf Normalverteilung getestet. Sofern dies gegeben war, wurde der Student’s t‑Test verwendet. Sofern dies nicht gegeben war, wurde der Mann-Whitney-U-Test verwendet. Die Daten wurden als Mittelwert ± Standardabweichung angegeben. Ein α‑Fehler <0,05 wurde als statistisch signifikant angesehen.

## Ergebnisse

### Allgemeine Patientendaten

**Figure 1:**
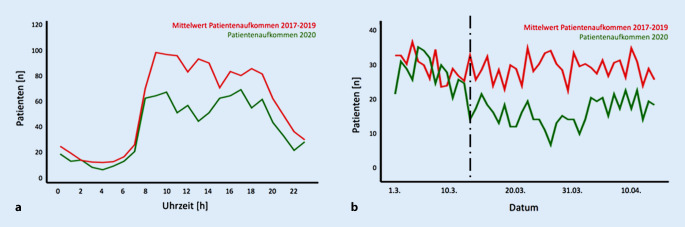

Es wurden insgesamt 4967 Patienten im Beobachtungszeitraum 01.03.–15.4. der Jahre 2017–2020 eingeschlossen. In den 46 Tagen des Beobachtungszeitraums wurden im Durchschnitt der 3 Vorjahre 1348 Patienten und pro Tag 29,3 Patienten vorstellig. Demgegenüber wurden im Jahr 2020 im gleichen Beobachtungszeitraum insgesamt 923 und pro Tag 20 Patienten vorstellig (*p* < 0,01). Das entspricht einem Rückgang von 425 Patienten auf 68,4 % des durchschnittlichen Vorjahrespatientenaufkommens. Analog dazu zeigte sich eine Reduktion der Vorstellungszahlen im Hinblick auf die Uhrzeit der Vorstellung (Abb. 1a). Das durchschnittliche Patientenalter war im Mittelwert der 3 Vorjahre 47 ± 26,1 Jahre und 2020 49,3 ± 25,8 Jahre. Eine Subgruppenanalyse für den Zeitraum vom 13.03–15.04. zeigt eine noch deutlichere Reduktion auf 16,9 Patienten/Tag von zuvor 29,2 Patienten/Tag im Vergleichszeitraum der Vorjahre.

Im Mittelwert der Vorjahre wurden im Vergleich zu 2020 311,5 (23,1 %) vs. 227 (24,6 %) stationär aufgenommen. Demzufolge verblieben 76,9 % im Vergleichszeitraum und 75,4 % im Jahr 2020 ambulant. Es wurden im Jahr 2020 33 polytraumatisierte Patienten beobachtet. Demgegenüber standen im Mittelwert der 3 Vorjahre 26,3 Polytraumata. Dies entspricht einer Zunahme um 21,3 %. Im Mittelwert 2017–2019 resultierten aus den beschriebenen Pathologien und Verletzungen 143 Operationen im Vergleich 136 Operationen im Jahr 2020. Das Mittel der summierten Operationszeit (Schnitt-Naht [Minuten]) der Jahre 2017–2019 betrug 18.971 min und unterschied sich nicht von den summierten Operationszeiten im Jahr 2020 mit 18.837 min (*p* = 0,72). Die mittlere Anzahl der stationären Tage von 2442 unterschied sich im Jahr 2020 mit 1172 Tagen (−52,1 %) (*p* < 0,01). Die Anzahl der Tage auf der ICU betrug im Mittel der Vorjahre 450 Tage und im Jahr 2020 mit 303 Tagen (−32,7 %) (*p* < 0,01) (Tab. 5).

**Table 5:** 

Unfallchirurgische/orthopädische Krankheitsbilder	Stat. P. 17–19 [*n*]	Stat. P. 20 [*n*]	OP-Kapazität 17–19 [min]	OP-Kapazität 20 [min]	Stat. A. 17–19 [d]	Stat. A 20 [d]	ICU 17–19 [Tage]	ICU 20 [Tage]
Verletzungen, Kopf [S00–S09]	59	52	1702	1929	341	304	143	109
Verletzungen des Halses [S10–S19]	7	9	710	1062	81	55	6	34
Verletzungen des Thorax [S20–S29]	22	13	1478	1804	202	22	45	38
Verletzungen des Abdomens, des Lumbosakralgelenks, der LWS und des Beckens [S30–S39]	17,3	18	831	1189	166	102	31	16
Verletzungen, Schulter/Oberarm [S40–S49]	21	7	283	2360	39	33	0	17
Verletzungen, Ellenbogengelenk/Unterarm [S50–S59]	20,3	11	1146	887	92	36	5	4
Verletzungen, Handgelenk/Hand [S60–S69]	30	2	0	105	3	2	0	0
Verletzungen der Hüfte und des Oberschenkels [S70–S79]	36,3	39	4022	3254	510	254	75	48
Verletzungen des Knies und des Unterschenkels [S80–S89]	31	25	4367	2076	292	113	39	0
Verletzungen der Knöchelregion und des Fußes [S90–S99]	24,6	4	1237	121	135	9	12	0
Summe, T‑Diagnosen	11	16	1842	1723	230	86	63	6
Summe, M‑Diagnosen	13	13	709	1249	179	68	28	4
Summe Diagnosen Sonstige	19	18	644	1078	172	88	3	27
*Summe Total*	*311,5*	*227*	*18.971*	*18.837*	*2442*	*1172*	*450*	*303*

*n* Anzahl, *min* Minuten, *d* Tage, *stat. P*. stationäre Patienten, *stat. A.* stationäre Aufenthaltsdauer, *ICU* Aufenthaltsdauer, Intensivstation

### Körperregionsspezifische Verletzungen

Verletzungen des Kopfes machten 2020 im Vergleich zu 2017–2019 mit 20,8 % vs. 20,3 % der Verletzungen den größten Anteil aus. Es folgten Verletzungen des Knies/Unterschenkels mit 9,9 % vs. 9,5 %, Verletzungen des Sprunggelenks/Fuß mit 8,7 % vs. 12,1 % und Verletzungen von Ellenbogen/Unterarm mit 8,4 % vs. 6,2 % (Tab. 1).

Im Mittelwert wurden im Beobachtungszeitraum 2020 insgesamt 260 Frakturen im Vergleich zu 2017–2019 mit 359,3 Frakturen gezählt. Dies entspricht einer Reduktion um 27,6 %. Dabei machten im Jahr 2020 im Vergleich zum Mittelwert der 3 Vorjahre Frakturen des Ellenbogens/Unterarms mit 21,2 % vs. 14,9 % (−6,3 %) den größten Anteil aus. Es folgten Frakturen von Hüfte/Oberschenkel 15,4 % vs. 9,9 % (−5,5 %) und Frakturen von Knie/Unterschenkel mit 13,9 % vs. 11,3 % (−2,6 %) (Tab. 2). Es wurden 31 geriatrische proximale Femurfrakturen bei Patienten älter als 70 Lebensjahre im Jahr 2020 beobachtet. Demgegenüber standen im Mittelwert der 3 Vorjahre 25 Patienten. Das durchschnittliche Alter aller Patienten mit proximalen Femurfrakturen stieg von 75,57 Jahre im Vergleichszeitraum der 3 Vorjahre auf 78,13 Jahre im Jahr 2020.

Im Mittelwert wurden im Beobachtungszeitraum 2020 im Vergleich zu 2017–2019 insgesamt 73 vs. 125,2 offene Wunden gezählt. Dabei machten offene Wunden des Kopfes mit 54,8 % vs. 52,3 % den größten Anteil aus. Es folgten Wunden von Knie/Unterschenkel mit 15,1 % vs. 10,1 % und Sprunggelenk/Fuß mit 6,8 % vs. 5,4 % (Tab. 3).

Insgesamt kam es zu einem Rückgang der dokumentierten Verletzungsmechanismen von 1255 im Mittelwert der 3 Vorjahre auf 933 im Jahr 2020 (−25,66 %). Im Beobachtungszeitraum 2020 wurden insgesamt 259 (27,8 %) Verletzungen im häuslichen Umfeld im Vergleich zum Mittelwert der Jahre 2017–2019 mit 267 (21,2 %) Verletzungen im häuslichen Umfeld beobachtet (+6,6 %). Analog dazu gab es einen Rückgang der Verletzungen bei sportlicher Aktivität von 164 (13,1 %) im Mittelwert der Jahre 2017–2019 auf 73 (7,8 %) im Jahr 2020 um −5,3 %. Es konnte lediglich eine Zunahme von 0,4 % im Bereich der häuslichen Gewalteinwirkung beobachtet werden (Tab. 4).

## Diskussion

In unserem Beobachtungszeitraum 2020 konnten wir im Vergleich zum Mittelwert des gleichen Beobachtungszeitraums 2017–2019 drei wesentliche Beobachtungen machen:

- Die Anzahl der täglich vorstellig werdenden Patienten ist von 29,3 auf 20 gefallen.
- Damit verbunden kam es zu einer Reduktion der Verletzungen und zu einer Verschiebung der Frakturhäufigkeit.
- Damit verbunden kam es zu einer Reduktion der Operationszahl sowie der absoluten Anzahl der stationären Tage und der Tage auf der ICU.

Ein schrittweiser Rückgang der Patientenzahlen in der Notaufnahme lässt sich ab dem 01.03.2020 mit dem Auftreten des ersten SARS-CoV‑2 positiv getesteten Patienten in der Region Hannover feststellen. Dem zugrunde liegt mutmaßlich eine in der Bevölkerung empfundene Sorge, sich im Krankenhaus mit dem Virus zu infizieren. Die absolute Reduktion der Patientenzahl in unserer Notaufnahme wird besonders mit Inkrafttreten des Beschlusses der Landesregierung vom 13.03.2020, bzw. in den darauffolgenden Tagen, deutlich (Abb. 1b). Hieraus erging mit dem 16.03.2020 u. a. die Schließung von Schulen und Universitäten, eine Einreisebeschränkung für 30 Tage und die Absage aller Veranstaltungen.

Im Vergleich zu den Vorjahren war im Jahr 2020 ein ähnliches Verletzungsmuster zu beobachten. Während sowohl im Jahr 2020 als auch im Vergleichszeitraum der 3 Vorjahre die prozentuellen Anteile der Kopfverletzungen mit 20 % und der der Unterschenkel‑/Knieverletzungen mit jeweils 9 % gleich blieben, war im Vergleichszeitraum 2017–2019 die Verletzung von Sprunggelenk/Fuß mit 12,1 % etwas stärker betroffen als im Jahr 2020 mit 8,7 %. Insgesamt scheint es hier zu einer gleichförmigen Reduktion der Verletzungszahlen ohne wesentliche Verschiebungen gekommen zu sein. Eine Reduktion der Verletzungen von Sprunggelenk/Fuß von 3,4 % war hier die auffälligste Entwicklung im Vergleich zum Mittelwert der Vorjahre.

Es fiel jedoch auf, dass im Hinblick auf die Frakturen im Jahr 2020 Verletzungen von Ellenbogen/Unterarm, Hüfte/Oberschenkel, Knie/Unterschenkel und Kopf häufiger vertreten waren als im Mittel der Vorjahre. Demgegenüber waren Frakturen von Handgelenk/Hand und Sprunggelenk/Fuß im Mittel der Vorjahre stärker repräsentiert als im Jahr 2020. Laut dem Sportreport 2017 der VBG machen Verletzungen von Sprunggelenk und Kopf den Großteil der Sportverletzungen aus [9]. Wenn wir betrachten, dass es in unserem Kollektiv zu einem Rückgang des Verletzungsmechanismus beim Sport um 5,3 % kam, mag diese proportionale Verschiebung des Verletzungsmusters erklärbar sein. Die relative Zunahme der Verletzungen im häuslichen Umfeld um 6,6 % mag die Zunahme der Unterarm- und hüftgelenknahen Frakturen erklären. Hier haben wir besonders bei den geriatrischen proximalen Femurfrakturen bei Patienten älter als 70 Lebensjahre eine Zunahme beobachtet. Wie Lohmann et al. bereits 2007 zeigen konnten, machen diese Frakturen zusammen mit den Humerusfrakturen den Großteil der geriatrischen Frakturen aus [10]. Es bleibt jedoch zu mutmaßen, ob diese proportionale Verschiebung der Frakturlokalisationen hierdurch erklärt werden kann, da das durchschnittliche Alter des Patientenkollektivs sich nicht wesentlich unterschied. Ferner konnte eine Zunahme der polytraumatisierten Patienten um gut 20 % beobachtet werden. Dies könnte an der Verlagerung der Schwerstverletztenversorgung von Grund- und Regelversorgern zu Maximalversorgern durch Reduktion der dortigen vorgehaltenen Ressourcen liegen. Eine separate Auswertung des präklinischen Meldebildes läuft zurzeit in unserer Klinik.

Betrachtet man die Wunden nach Körperregionen, so sind hier keine wesentlichen Unterschiede sichtbar. Dies scheint sich als ein Abbild der Gesamtverletzungsmuster darzustellen.

Im Hinblick auf den dokumentierten Verletzungsmechanismus war lediglich eine Zunahme der häuslichen Gewalt um 0,4 % im Jahr 2020 festzustellen. Dies ist überraschend, da besonders medial mit einer deutlichen Zunahme gerechnet wurde. Möglicherweise mag dem beobachteten Wert eine größere Dunkelziffer zugrunde liegen, wodurch die Werte nur bedingt der Realität entsprechen.

Wir konnten in unserer Untersuchung ein ähnliches Verletzungsaufkommen beobachten wie in der von Biberthaler et al. beobachteten Studie [6]. Während die Kopfverletzungen in unserer und der genannten Studien den größten Anteil ausmachen, waren bei Biberthaler et al. die Verletzungen von Unterarm und Hand am zweithäufigsten vertreten; und in unserer Studie waren es die Verletzungen von Knie und Unterschenkel. Die Vergleichbarkeit ist sicherlich eingeschränkt, da wir nur einen Zeitraum von 6 Wochen in einer Klinik betrachteten und in der genannten Arbeit über ein Jahr der Großraum München beobachtet wurde.

Obwohl es im Jahr 2020 zu einer deutlichen Reduktion von 84 Patienten (27,1 %) der stationären Aufnahmen kam, kam es im Vergleich zum Vergleichszeitraum der 3 Vorjahre zu keinem signifikanten Rückgang der Operationsminuten (18.971 vs. 18.837 min). Ob dieser Beobachtung einer Zunahme der Verletzungsschwere bei rückläufigen Operationszahlen zugrunde liegt, bleibt unklar. Zu mutmaßen ist, dass die gleichbleibende Dauer der Operationsminuten durch die Zunahme der polytraumatisierten Patienten erklärbar sein kann.

Die Reduktion der stationären Aufnahmen war auch mit einer Reduktion der stationären Tage verbunden (2442 vs. 1172 [−52,1 %]). Dies hat sicherlich zu der gewünschten Entlastung der Klinikstrukturen beigetragen. Auch die für COVID-19-Patienten wichtige Intensivkapazität wurde deutlich eingespart. So kam es 2020 zu einer Summe von 303 Tagen auf der Intensivstation im Vergleich zu 450 Tagen im Vergleichszeitraum der 3 Vorjahre. Dies entspricht einer Reduktion um 32,7 %. Wir können somit feststellen, dass, vermutlich durch die politisch getroffenen Maßnahmen bedingt, es auch im Bereich der Unfallchirurgie zu einer Reduktion des Notaufnahmeaufkommens gekommen ist. Zudem ist es im Jahr 2020 zu Einsparungen der Normalstationskapazität um 71,4 % sowie der ICU-Kapazität um 61,4 %, im Vergleich zu den Mittelwerten der 3 Vorjahre, gekommen.

### Limitation

Auf der Basis der gewonnenen Daten lässt sich im Jahr 2020 eine Reduktion der unfallchirurgischen Fallzahlen – im Vergleich zum Mittelwert der 3 Vorjahre – feststellen. Eine kausale Korrelation mit den getroffenen politischen Maßnahmen lässt sich jedoch nicht sicher herleiten. Die subjektiv empfundene Angst der Ansteckung mit COVID-19 ist möglicherweise ein Faktor, der die Vorstellung von Patienten in der Notaufnahme beeinflusst. Hierdurch mag es zu einem Zerrbild der real stattgefunden Verletzungen gekommen sein. Obwohl unsere Daten der von Biberthaler et al. publizierten Verteilung des Verletzungsmusters ähneln, scheint hier eine Übertragbarkeit auf weitere Kliniken und Städte auf der doch relativ geringen Fallzahl an nur einem Standort und über einen limitierten Zeitraum, reduziert. Wir sind jedoch der Meinung, dass die hier gewonnenen Daten verdeutlichen, dass es insgesamt zu einem Rückgang der Klinikbelastung durch unfallchirurgische Patienten gekommen ist. Hierdurch sind Ressourcen für die Betreuung von COVID-19-Patienten frei geworden.

## Fazit

Durch politische Vorgaben wurde versucht, die Infektionszahlen der COVID-19-Pandemie und ebenso die Belastung des Gesundheitssystems zu reduzieren. Wir haben in unserer Klinik 4967 Patienten evaluiert, die sich in unserer Notaufnahme vorgestellt haben und entweder ambulant oder stationär verblieben sind. Wir konnten mit unserer Arbeit den Einfluss des unfallchirurgischen Patientenaufkommens auf die Gesamtbelastung der Klinik im Vergleich zu den 3 Vorjahren beschreiben. Im Jahr 2020 kam es im Vergleich zu den 3 Vorjahren im Zeitraum vom 01.03.–15.04. zu einer Reduktion des Patientenaufkommens in unserer Notaufnahme. Damit verbunden waren Reduktionen:

- der stationären Aufnahmen um 27,1 %,
- der stationären Tage um 52,1 %,
- der benötigten ICU-Kapazität um 32,7 %.

Wir schlussfolgern hieraus, dass durch die getroffenen Maßnahmen eine direkte Entlastung des Gesundheitssystems – auch durch Reduktion des unfallchirurgischen Patientenaufkommens – beobachtet werden kann. Die gewonnenen Daten können für etwaige spätere Krisensituationen genutzt werden und geben Anhalt für mögliche Ressourcenverteilungen.
